# Galectin-3 Mediates Heme-Induced Multi-Organ Dysfunction by Modulating the Splenic Immune Microenvironment

**DOI:** 10.3390/diseases14050161

**Published:** 2026-05-06

**Authors:** Mirjana Milinkovic, Marija Milovanovic, Jelena Milovanovic

**Affiliations:** 1 Institute for Transfusiology and Hemobiology, Military Medical Academy, 11000 Belgrade, Serbia; mirjanamilinkovic4@gmail.com; 2 Center for Harm Reduction of Biological and Chemical Hazards, Faculty of Medical Sciences, University of Kragujevac, Svetozara Markovića 69, 34000 Kragujevac, Serbia; marijaposta@gmail.com; 3 Department of Microbiology and Immunology, Faculty of Medical Sciences, University of Kragujevac, Svetozara Markovića 69, 34000 Kragujevac, Serbia; 4 Department of Histology and Embryology, Faculty of Medical Sciences, University of Kragujevac, 34000 Kragujevac, Serbia

**Keywords:** galectin-3, intravascular hemolysis, systemic inflammation, T cell polarization, multi-organ injury, regulatory T cells

## Abstract

Background/Objectives: Acute intravascular hemolysis is associated with the release of labile heme, which contributes to systemic inflammation and organ dysfunction. Galectin-3 (Gal-3) is a known modulator of inflammatory responses. However, its specific role in heme-induced organ injury remains to be fully elucidated. Methods: We used a phenylhydrazine (PHZ)-induced model of acute hemolysis in wild-type (WT) and Gal-3 knockout (KO) mice to investigate the influence of Gal-3 on tissue alterations and the inflammatory response. Results: Despite equivalent levels of hemolysis and anemia in both genotypes, Gal-3 deficiency was associated with reduced injury in the liver, kidneys, and pancreas. In WT mice, Gal-3 was associated with a pro-inflammatory splenic microenvironment. Conversely, Gal-3 KO mice exhibited a shift toward an immunoregulatory phenotype, characterized by an increased frequency of CD4 + CD25 + FoxP3+ regulatory T cells and IL-10+ macrophages. This shift correlated with preserved organ architecture and a more controlled inflammatory profile. Conclusions: Our findings suggest that Gal-3 may act as a mediator of heme-induced systemic inflammation. By influencing the splenic immune microenvironment and promoting a regulatory phenotype, the absence of Gal-3 appears to alleviate multi-organ stress, suggesting its potential as a target for modulating complications during acute hemolytic crises.

## 1. Introduction

Intravascular hemolysis is a critical pathological process characterized by the premature destruction of erythrocytes within the circulation, leading to the massive release of hemoglobin into the plasma. This phenomenon is a hallmark of numerous clinical conditions, ranging from hereditary hemoglobinopathies like sickle cell disease and thalassemias to acquired states such as severe transfusion reactions, mechanical trauma from prosthetic heart valves, and certain systemic infections [1,2,3]. Our previous research has established Galectin-3 (Gal-3) as a pivotal regulator of innate immunity, particularly in the context of inflammasome activation and organ-specific inflammatory diseases [4]. While the immediate consequence of hemolysis is the loss of oxygen-carrying capacity, the most devastating clinical outcomes are often driven by the systemic inflammatory response syndrome (SIRS) and subsequent multi-organ dysfunction syndrome (MODS) [2].

The primary culprit behind this systemic inflammatory response is cell-free hemoglobin and its immediate oxidation product, labile heme [5,6]. Under physiological conditions, haptoglobin and hemopexin safely sequester these pigments. However, during massive hemolytic events, these protective systems become saturated [2,7,8]. Free heme acts as a potent pro-oxidant and a damage-associated molecular pattern (DAMP), capable of triggering sterile inflammation by activating the innate immune system [9,10]. Specifically, heme has been shown to bind to and activate the Toll-like receptor 4 (TLR4) signaling pathway, leading to the nuclear translocation of NFκB and the subsequent production of pro-inflammatory cytokines such as TNF-α, IL-6, and IL-1β [10,11,12].

Despite the established role of heme in promoting tissue injury, the exact molecular amplifiers that convert localized hemolytic stress into systemic organ failure remain to be fully elucidated. Given that heme-induced inflammation relies on the orchestration of multiple DAMPs, Gal-3 emerges as a compelling candidate for regulating these pathways. Recent evidence, including our own studies on autoimmune and infectious liver diseases, has positioned Gal-3 as a central player in the regulation of innate and adaptive immune responses [12,13,14]. Gal-3 is uniquely characterized by its chimeric structure, allowing it to form pentamers that can cross-link cell surface receptors and stabilize signaling complexes [15,16]. It is widely expressed in myeloid cells, including macrophages and dendritic cells, and its expression is markedly upregulated in response to tissue injury and oxidative stress [8,17].

Our previous research has demonstrated that Gal-3 is essential for the full-scale activation of the NLRP3 inflammasome and is an important driver of inflammation in various organ-specific models, including immune-mediated hepatitis and acute pancreatitis [4,13]. Crucially, Gal-3 has been identified as an “alarmin” or secondary DAMP that can synergize with other pro-inflammatory stimuli to exacerbate tissue damage [14,17,18]. However, its specific role in the context of acute intravascular hemolysis, where it may interact with the heme-TLR4 axis, has recently been hypothesized by our group [19].

Furthermore, the impact of hemolysis extends beyond acute tissue necrosis. The spleen, as the primary site for the clearance of damaged erythrocytes, must orchestrate a rapid transition toward stress erythropoiesis to restore hematological homeostasis [20,21]. Emerging data suggest that the pro-inflammatory environment generated during a hemolytic crisis may act as a “molecular brake” on erythroid progenitor expansion, although the role of Gal-3 in this inhibitory process remains unexplored [22,23,24].

In this study, we employed a well-established model of phenylhydrazine (PHZ)-induced acute intravascular hemolysis in both wild-type (WT) and Galectin-3 knockout (KO) mice [8,25]. By utilizing a global knockout approach, we aimed to determine whether Gal-3 deficiency could mitigate the systemic inflammatory surge and protect vital organs, specifically the liver, kidneys, and pancreas, from heme-mediated inflammatory injury. Our findings identify Gal-3 as a significant contributor of hemolysis-induced MODS and a critical regulator of the Th1/Th17 versus Treg balance [19].

## 2. Materials and Methods

### 2.1. Animals and Intravascular Hemolysis Induction

Male C57BL/6 wild-type (WT) and Galectin-3 knockout (Gal-3 KO) mice, aged 12 to 16 weeks, weighing 22–27 g, were used in this study. The founder Gal-3 KO mice were kindly provided by D.K. Hsu and F.T. Liu from the University of California, Davis (Davis, CA, USA). For all experiments, Gal-3 KO mice and age- and sex-matched wild-type (WT) C57BL/6 control mice were obtained from the same source and maintained under identical environmental conditions in our facility. The mice were maintained under standard laboratory conditions in the Animal Facility of the Faculty of Medical Sciences, University of Kragujevac (12 h light/dark cycle, temperature 22 ± 2 °C) with ad libitum access to food and water. All experimental procedures were approved by the Ethics Committee of the Faculty of Medical Sciences, University of Kragujevac (protocol number 01-14241), and were conducted in strict accordance with the National Institutes of Health Guide for the Care and Use of Laboratory Animals. The study was conducted in accordance with the ARRIVE guidelines for the design, analysis, and reporting of animal experiments.

A total of 54 mice were divided into four groups: (1) C57BL/6 WT mice which received an intraperitoneal injection of phenylhydrazine (PHZ) (Cat. Number P26252, Sigma Aldrich, St. Louis, MO, USA), (2) C57BL/6 WT mice untreated, (3) C57BL/6 Gal-3 KO mice which received i.p. PHZ, (4) C57BL/6 Gal-3 KO mice, untreated. Acute intravascular hemolysis was induced by a single intraperitoneal injection of freshly prepared phenylhydrazine at a dose of 75 mg/kg, dissolved in 200 µL of physiological saline. To ensure the reproducibility of our findings, experiments were conducted in two independent series. The first series (*n* = 4–6 per group) focused on peripheral blood hematology and flow cytometric analysis of splenic immune populations. The second series (*n* = 8–9 per group) was dedicated to the assessment of serum biochemical markers and histopathological evaluation of target organs. All experimental procedures, including PHZ dosing and time points, were identical across both series.

Mice were sacrificed 48 h post-application via cervical dislocation, in a method performed by highly trained personnel in strict accordance with the institutional ethical guidelines to ensure a rapid and painless procedure.

Blood was collected for blood and biochemical analyses. Liver, spleen, pancreatic and kidney tissues were harvested for histological examination. Additionally, spleen mononuclear cells (MNCs) were isolated for comprehensive flow cytometric analysis.

### 2.2. Blood Analysis

Blood samples were immediately drawn by aortic puncture of each mouse. The hematological parameters were measured by an automatic hematological assay analyzer (Sysmex SF-3000 Hematology Analyzer, Sysmex Corporation, Kobe, Japan).

### 2.3. Biochemical Analyses

Serum levels of alanine aminotransferase (ALT), amylase, creatinine and urea were measured 48 h post-PHZ administration. Analysis was performed via spectrophotometry using an automated biochemistry analyzer (Olympus AU 400; Olympus Diagnostica GMBH, Hamburg, Germany).

### 2.4. Histological Evaluation

Liver, kidney, spleen, and pancreas tissues were harvested and fixed in 4% paraformaldehyde. Following fixation, tissues were embedded in paraffin, sectioned at a thickness of 3 µm, and stained with hematoxylin and eosin (H&E) for histological examination. The slides were analyzed using a light microscope (Leica DM2500, Wetzlar, Germany) equipped with a Leica Flexacam i5 digital camera.

The extent of hepatic injury was blindly assessed by two independent researchers using a semi-quantitative scoring system. The evaluation focused on three primary histological features observed in response to hemolytic stress: 1. vascular congestion: graded from 0 to 3 based on the degree of sinusoidal and venous dilatation; 2. inflammatory infiltration: assessed by counting the number of inflammatory foci per high-power field (hpf), where 0 = none, 1 ≤ foci, 2 = 2–4 foci, and 3 ≥ foci; and 3. hepatocellular damage: evaluated by the presence of cellular swelling (ballooning) and apoptotic bodies, graded on a scale of 0 (absent) to 3 (extensive). The total histological score was calculated as the sum of these individual scores to represent the overall severity of hepatic tissue damage [26].

Pancreatic tissue sections were evaluated blindly by two independent investigators using a semi-quantitative scoring system [27]. The following parameters were graded on a scale from 0 to 3: 1. interstitial edema: 0 = absent, 1 = expanded intercellular spaces, 2 = expanded interlobular spaces, 3 = integrated acini separated by expanded connective tissue; 2. inflammatory infiltration: 0 = absent, 1 = 1–20 inflammatory cells/HPF, 2 = 21–50 inflammatory cells/HPF, 3 ≥50 inflammatory cells/hpf; and 3. acinar cell vacuolization: 0 = absent, 1 = <20% of acinar cells involved, 2 = 21–50% of cells involved, 3 = >50% of cells involved. The final histological score was expressed as the sum of the individual scores for each animal [27].

Renal tubular injury was assessed on H&E-stained sections using a semi-quantitative scale based on the percentage of affected tubules showing luminal debris, loss of brush border, or cytoplasmic vacuolization. The scoring system was as follows: 0, no injury; 1, <10% of tubules affected; 2, 11–25% affected; 3, 26–50% affected; and 4, >50% of tubules showing signs of injury. For each animal, 10–15 randomly selected cortical fields were evaluated at 200× magnification by an investigator blinded to the experimental groups [28].

### 2.5. Phenotypic Characterization of Splenocytes

Mononuclear cells were isolated from the spleen, using previously described protocols. The isolated cells were analyzed by flow cytometry using fluorochrome-conjugated monoclonal antibodies against the following markers: F4/80, CD11c, CD25, CD4, CD8, FoxP3, TNF-α, IL-10, IL-6, IL-1β, IFN-γ and IL-17 (BD Biosciences). For intracellular staining of cytokines, after staining surface markers, cells were incubated for 4 h at 37 °C in the presence of 50 ng/mL phorbol 12-myristate 13-acetate (PMA) (P8139, Sigma-Aldrich, St. Louis, MO, USA), 1 μg/mL ionomycin (I0634, Sigma-Aldrich, St. Louis, MO, USA) and Golgi Stop (Cat. Number 554724, BD Biosciences, San Jose, CA, USA). After incubation with PMA and ionomycin, cells were fixed and permeabilized using a BD Cytofix/Cytoperm kit (Cat. Number 554714, BD Biosciences, San Jose, CA, USA). After fixation and permeabilization, cells were incubated with anti-cytokine antibodies. To ensure accurate gating and define positive populations, Fluorescence Minus One (FMO) controls were utilized for all cytokines and FoxP3. Expression of cell surface and intracellular antigens was analyzed using a FACSCalibur Flow Cytometer (BD Biosciences, San Jose, CA, USA). Flow cytometric analysis was conducted with FlowJo Software V9 (Tree Star, Phoenix, AZ, USA).

### 2.6. Statistical Analysis

Statistical analysis was performed using IBM SPSS Statistics v22.0. The normality of the data distribution was assessed using the Shapiro–Wilk test, while the homogeneity of variances was evaluated via Levene’s test. Data are presented as mean values ± standard deviation (SD) or, where appropriate, as mean differences (MD) with 95% confidence intervals (95% CI) to indicate effect magnitude and precision.

For comparisons among the four experimental groups, a one-way analysis of variance (ANOVA) was applied for normally distributed data. When a significant overall difference was detected, post hoc pairwise comparisons were conducted using Bonferroni correction to maintain a stringent control over the Type I error rate during multiple comparisons. All data met the assumptions for parametric analysis. Statistical significance was defined as *p* < 0.05, with exact *p*-values reported throughout the manuscript to provide full transparency.

## 3. Results

### 3.1. Gal-3 Deficiency Is Associated with Attenuated Systemic Leukocytosis Despite Equivalent PHZ-Induced Hemolysis

**Figure 1:**
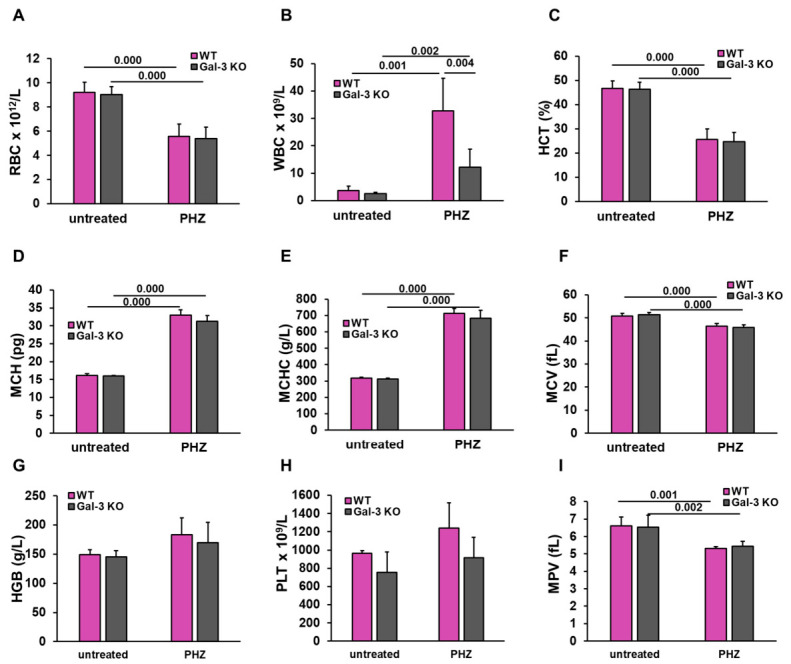
Galectin-3 deficiency does not affect the severity of phenylhydrazine-induced hemolytic anemia but attenuates resulting systemic leukocytosis. Peripheral blood parameters were analyzed to assess the severity of anemia and systemic cellular response 48 h following PHZ administration, including (**A**) red blood cell (RBC) count, (**B**) white blood cell (WBC) count, (**C**) hematocrit (HCT), (**D**) mean corpuscular hemoglobin (MCH), (**E**) mean corpuscular hemoglobin concentration (MCHC), (**F**) mean corpuscular volume (MCV), (**G**) hemoglobin (HGB) concentration, (**H**) platelet (PLT) count, and (**I**) mean platelet volume (MPV). Data are presented as mean + SD (*n* = 4 per untreated group; *n* = 6 per PHZ-treated group). Statistical significance was determined using one-way ANOVA with a Bonferroni post hoc test, with exact *p*-values indicated above the comparison bars.

To investigate the role of Gal-3 in the development of acute hemolytic anemia, we first analyzed the peripheral blood parameters in wild-type and Gal-3 KO mice following PHZ administration. Our initial hematological screening confirmed the successful induction of severe erythroid depletion across both genotypes. Baseline levels of red blood cell (RBC) counts, hemoglobin (HGB), hematocrit (HCT), and mean corpuscular volume (MCV) were comparable between WT and Gal-3 KO mice. Administration of PHZ led to a significant reduction in blood cell count (RBC) (Figure 1A), hematocrit (HCT) (Figure 1C), and mean corpuscular volume (MCV) (Figure 1F) in both WT and Gal-3 KO groups compared to untreated controls, indicating that the primary hemolytic insult occurs independently of Gal-3 expression. Both groups exhibited a sharp increase in mean corpuscular hemoglobin (MCH) (Figure 1D) and mean corpuscular hemoglobin concentration (MCHC) (Figure 1E) following PHZ treatment, reflecting the compensatory erythropoietic shifts and the presence of circulating hemoglobin. While hemoglobin levels showed a slight upward trend in PHZ-treated groups (Figure 1G), likely due to the presence of free plasma hemoglobin, no significant differences were observed between WT and Gal-3 KO mice.

These data suggest that the primary hemolytic insult and the resulting anemia occur independently of Galectin-3 expression.

Crucially, the systemic response to hemolysis differed significantly between the two genotypes. While PHZ triggered a substantial increase in the total white blood cell (WBC) count in both groups, the magnitude of this leukocytosis was significantly attenuated in Gal-3 KO mice compared to their WT counterparts (Figure 1B; *p* = 0.0040). The mean platelet volume (MPV) decreased in both groups following the hemolytic insult (Figure 1I), with no genotype-specific differences noted.

These data suggest that while Gal-3 is not required for the initial chemical lysis of erythrocytes, it may act as a modulator of the subsequent systemic inflammatory response and hematopoietic remodeling, characterized by massive leukocyte mobilization and altered platelet dynamics, setting the stage for further tissue-specific inflammatory infiltration.

### 3.2. Gal-3 Deficiency Is Associated with Attenuated Heme-Induced Hepatic and Pancreatic Damage

**Figure 2:**
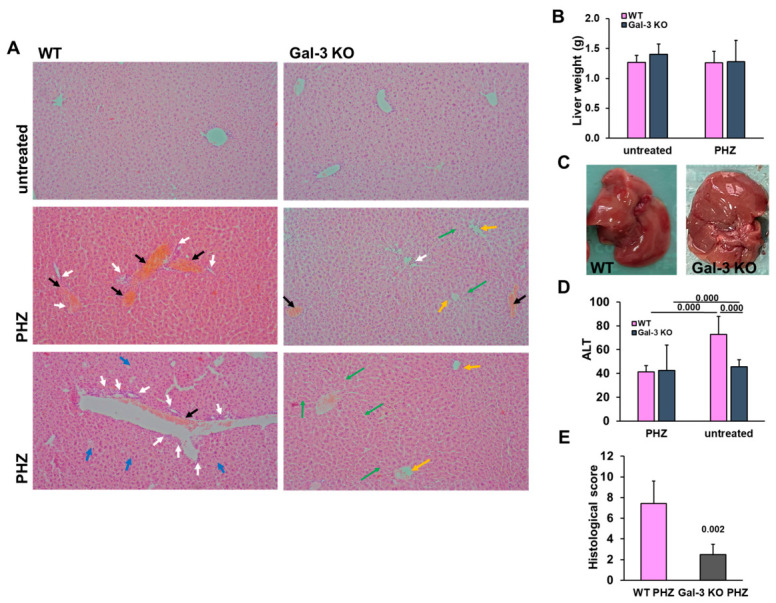
Galectin-3 deficiency protects against heme-induced acute liver injury. (**A**) Representative microphotographs of H&E-stained liver sections, magnification: 200×. In PHZ-treated WT mice, black arrows indicate marked vascular congestion, white arrows highlight focal inflammatory infiltrates, and blue arrows point to areas of hepatocellular damage/apoptosis. In contrast, Gal-3 KO mice displayed preserved hepatic architecture, with green arrows indicating normal sinusoidal patterns and yellow arrows marking the absence of vascular congestion. (**B**) Liver weight analysis across experimental groups, showing no significant differences in absolute liver mass. (**C**) Gross morphological appearance of livers from PHZ-treated WT and Gal-3 KO mice. (**D**) Serum alanine aminotransferase (ALT) levels as a biochemical marker of hepatocellular injury. (**E**) Semi-quantitative histological injury score. Data are expressed as mean ± SD (*n* = 8 animals per group for untreated mice and *n* = 9 mice per group for PHZ groups). Statistical significance for panels (**B**,**D**) was determined using one-way ANOVA with a Bonferroni post hoc test; for panel (**E**), statistical significance was determined using Student’s *t*-test, with exact *p*-values indicated.

To assess the systemic impact of acute hemolysis on peripheral organs, we evaluated hepatic morphology and biochemical markers of liver damage in both WT and Gal-3 KO mice. Gross morphological examination and liver weight analysis showed no significant differences in absolute liver mass between the genotypes or treatment groups (Figure 2B,C), indicating that the injury at this acute stage is characterized by cellular and microvascular dysfunction rather than gross organ hypertrophy. Histopathological analysis of H&E-stained liver sections underscored the protective effect of Gal-3 deletion. While marked vascular congestion, hepatocellular swelling, and inflammatory infiltrates were noticed in the livers of WT mice treated with PHZ, Gal-3 KO mice showed preserved lobular architecture and significantly reduced tissue damage (Figure 2A). To further quantify the observed morphological changes, we applied a semi-quantitative scoring system to evaluate hepatic tissue damage. In line with the biochemical findings, WT mice exhibited a high liver injury score (5.89 ± 2.15) following PHZ administration, whereas Gal-3 deficiency significantly attenuated the severity of damage, resulting in a lower histological score (2.89 ± 1.27; *p* = 0.002) (Figure 2E).

These morphological findings were biochemically validated by measuring ALT levels. While PHZ administration significantly elevated ALT in WT mice compared to untreated controls, this increase was significantly attenuated in Gal-3 KO mice (*p* = 0.000 vs. PHZ-treated WT) (Figure 2D). Collectively, these data demonstrate that the absence of Gal-3 provides substantial protection against heme-induced hepatotoxicity, highlighting its role as a key mediator of inflammatory damage during intravascular hemolysis.

**Figure 3:**
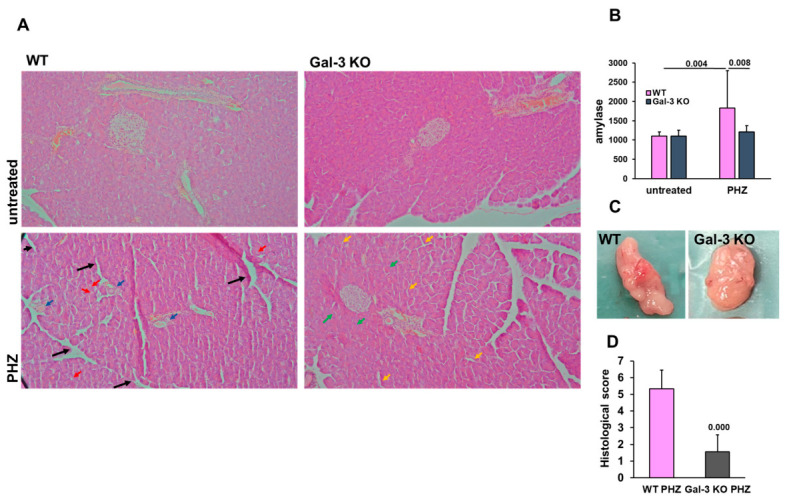
Galectin-3 deficiency mitigates heme-induced acute pancreatic injury. (**A**) Representative H&E-stained sections of pancreatic tissue, magnification: 200×. In PHZ-treated WT mice ((**A**), **bottom left**), black arrows indicate extensive interstitial edema, blue arrows highlight perivascular inflammatory infiltrates, and red arrows point to cytoplasmic vacuolization within acinar cells. In contrast, PHZ-treated Gal-3 KO mice show significantly preserved acinar architecture with dense cell populations (green arrows) and minimal evidence of interstitial expansion (yellow arrows). (**B**) Serum amylase activity. (**C**) Gross morphological appearance of the pancreas. (**D**) Semi-quantitative histological injury score. Data are expressed as mean + SD (*n* = 8 animals per group for untreated mice and *n* = 9 mice per group for PHZ groups). Statistical significance for panel (**B**) was determined using one-way ANOVA with a Bonferroni post hoc test; for panel (**D**), statistical significance was determined using Student’s *t*-test, with exact *p*-values indicated.

To explore whether the inflammatory response triggered by PHZ-induced hemolysis extends to the pancreas, we evaluated pancreatic morphology and biochemical markers of injury. Gross examination suggested that the pancreas in WT mice appeared more hyperemic following the hemolytic insult, while this appearance was less pronounced in Gal-3 KO mice (Figure 3C). Microscopic analysis of H&E-stained sections revealed that PHZ-treated WT mice developed histological features consistent with acute pancreatic stress, including interstitial edema, focal cytoplasmic vacuolization of acinar cells, and scattered perivascular inflammatory infiltrates (Figure 3A). These changes were quantified using a semi-quantitative scoring system. Notably, Gal-3 KO mice exhibited a significantly lower histological injury score compared to WT mice (*p* = 0.000), suggesting a higher degree of preserved acinar architecture in the absence of Gal-3 (Figure 3D). In support of these morphological findings, serum amylase levels were measured as a biochemical indicator of pancreatic involvement. PHZ administration resulted in a significant increase in amylase activity in WT mice compared to untreated controls (*p* = 0.004). Interestingly, this elevation was significantly attenuated in Gal-3 KO mice (*p* = 0.008), with values remaining closer to baseline levels (Figure 3B). Together, these data suggest that Gal-3 may participate in the development of secondary organ damage following acute hemolysis. Our findings indicate that the detrimental effects of the heme-mediated inflammatory cascade may extend to the pancreas, and that Gal-3 deficiency correlates with a more favorable outcome in this organ.

### 3.3. Gal-3 Deficiency Is Associated with Reduced Renal Stress Following Acute Hemolysis

**Figure 4:**
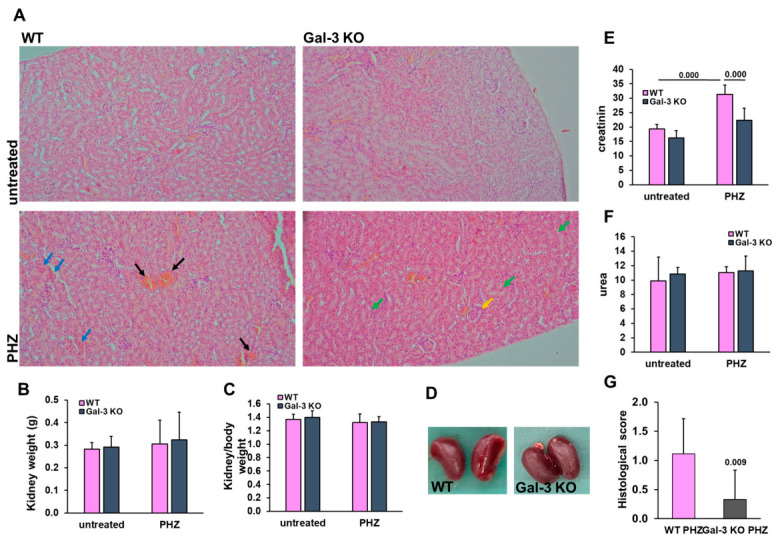
Galectin-3 deficiency is associated with reduced heme-induced renal stress. (**A**) Representative H&E-stained kidney sections, magnification: 200×. Representative H&E-stained sections of kidney cortex. In PHZ-treated WT mice, black arrows highlight areas of distinct vascular congestion, while blue arrows indicate occasional cellular debris within the tubular lumina, consistent with mild acute tubular stress. Conversely, Gal-3 KO mice displayed a more preserved renal morphology with intact tubular structures (green arrows) and minimal histological alterations (yellow arrows). (**B**) Absolute kidney weight and (**C**) kidney-to-body weight ratio. (**D**) Gross morphology of the kidneys. (**E**) Serum creatinine levels. (**F**) Serum urea levels across experimental groups. (**G**) Semi-quantitative histological injury score (Tubular Injury Score). Data are expressed as mean ± SD (*n* = 8 for untreated; *n* = 9 for PHZ groups). Statistical significance was determined using one-way ANOVA with a Bonferroni post hoc test for panels (**E**,**F**) and Student’s *t*-test for panel (**G**).

To further evaluate the extent of multi-organ involvement during acute hemolysis, we examined renal morphology and functional markers in both WT and Gal-3 KO mice. Microscopic analysis of H&E-stained kidney sections from PHZ-treated WT mice revealed signs of acute tubular stress, characterized by mild vascular congestion and occasional cellular debris within the tubular lumina (Figure 4A). In contrast, renal architecture in Gal-3 KO mice appeared largely preserved, showing significantly lower histological injury scores compared to WT counterparts (Figure 4G). Absolute kidney weights and kidney-to-body weight ratios did not show significant differences between genotypes or treatment groups (Figure 4B,C). Gross morphological inspection similarly revealed no overt macroscopic abnormalities, indicating that the injury at this time point is primarily functional and microstructural (Figure 4D). To assess renal function, serum levels of creatinine and urea were measured. PHZ administration resulted in a significant elevation of serum creatinine levels in WT mice compared to untreated controls (*p* = 0.000; Figure 4E). Notably, this increase was significantly attenuated in Gal-3 KO mice (*p* = 0.000 vs. WT post-PHZ), indicating a relative preservation of glomerular filtration capacity in the absence of Gal-3. Interestingly, serum urea levels (Figure 4F) remained relatively stable across all groups, suggesting that creatinine is a more sensitive early marker of acute renal stress in this specific model. Collectively, these findings suggest that Gal-3 deficiency correlates with a reduction in both the functional and histological manifestations of renal involvement following a hemolytic insult.

### 3.4. Gal-3 Deficiency Is Associated with Enhanced Splenic Expansion During Acute Hemolysis

**Figure 5:**
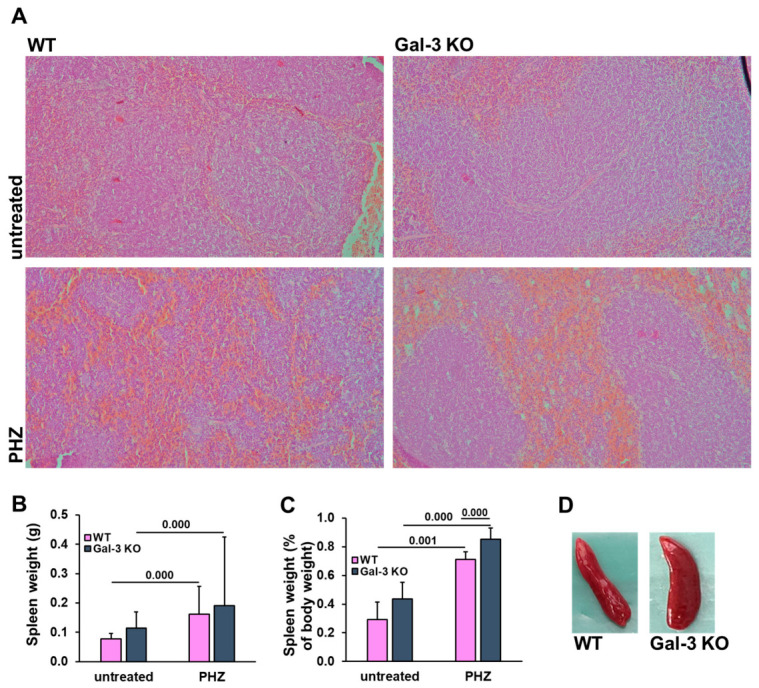
Galectin-3 deficiency is associated with enhanced splenic expansion and cellularity following acute hemolysis. (**A**) Representative H&E-stained sections of the spleen, magnification: 200×. (**B**) Absolute spleen weight. (**C**) Spleen weight as a percentage of body weight. (**D**) Gross morphological appearance of the spleens, highlighting the marked organ expansion in PHZ-treated groups. (Data are expressed as mean + SD (*n* = 8 animals per group for untreated mice and *n* = 9 mice per group for PHZ groups. Statistical significance was determined using one-way ANOVA with a Bonferroni post hoc test. Exact *p*-values for significant differences between specific groups are indicated above the respective comparison brackets.

Given the central role of the spleen in erythrocyte filtration and extramedullary erythropoiesis, we characterized the splenic response to PHZ-induced stress in both genotypes. Microscopic examination of splenic architecture revealed significant expansion of the red pulp in both WT and Gal-3 KO mice post-PHZ administration (Figure 5A). This architectural remodeling is consistent with the sequestration of damaged erythrocytes and the induction of stress erythropoiesis. PHZ treatment induced a significant increase in both absolute spleen weight and the spleen-to-body weight ratio in both genotypes (Figure 5B,C). Interestingly, while both groups exhibited marked splenomegaly, Gal-3 KO mice displayed a significantly more pronounced increase (in splenic mass relative to body weight compared to WT counterparts (Figure 5C; *p* = 0.000). This suggests a potentially distinct compensatory dynamic or altered cellular recruitment within the splenic niche in the absence of Gal-3.

The observed increase in splenic mass was further reflected in total splenocyte counts (Table 1). Both groups showed a significant increase in total cellularity following the hemolytic insult. However, the Gal-3 KO group exhibited a notable trend toward higher total splenocyte numbers (around 30 × 10^6^ in Gal-3 KO vs. 20 × 10^6^ in WT). These findings indicate that Gal-3 deficiency may influence the recruitment or local proliferation of immune and erythroid progenitor cells during the acute phase of hemolysis.

### 3.5. Galectin-3 Deletion Blunts the Pro-Inflammatory Activation and Cytokine Release of Splenic CD11c+ Dendritic Cells Post Hemolysis

**Figure 6:**
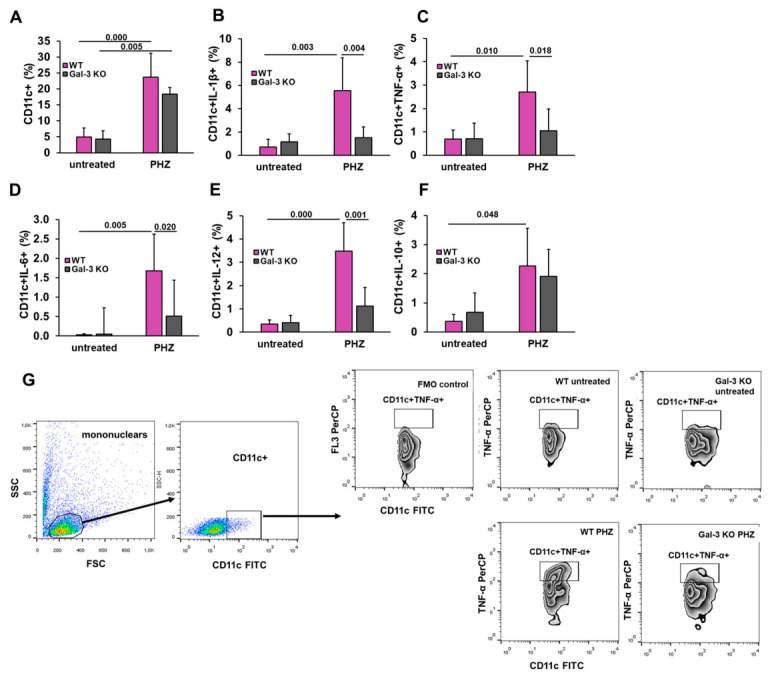
Galectin-3 deficiency prevents the pro-inflammatory activation of splenic dendritic cells following acute hemolysis. Flow cytometric analysis of splenic CD11c+ dendritic cells was performed 48 h post-PHZ administration. (**A**) Total frequency of CD11c+ dendritic cells in the spleen. The following panels represent the percentage of CD11c+ DCs expressing key cytokines: (**B**) IL-1β, (**C**) TNF-α, (**D**) IL-6, (**E**) IL-12, and (**F**) IL-10. (**G**) Gating strategy and representative flow cytometry plots. The gating sequence illustrates the identification of lymphocytes, followed by CD11c+ selection and representative plots for intracellular TNF-α staining in all experimental groups. A Fluorescence Minus One (FMO) control was used to define the gating cutoffs for cytokine-positive populations. Data are expressed as mean + SD. Sample sizes for each experimental group are as follows: untreated WT (*n* = 4), untreated Gal-3 KO (*n* = 4), PHZ-treated WT (*n* = 6), and PHZ-treated Gal-3 KO (*n* = 6). Statistical significance was determined using one-way ANOVA followed by Bonferroni post hoc test for multiple comparisons. Exact *p*-values for significant differences between specific groups are indicated above the respective comparison brackets.

To elucidate the cellular mechanisms underlying the observed systemic inflammation, we performed flow cytometric analysis of splenic CD11c+ dendritic cells and evaluated their intracellular cytokine expression following PHZ administration. PHZ administration triggered a significant expansion of the dendritic cell population, as evidenced by a marked increase in the percentage of CD11c+ cells in both WT and Gal-3 KO genotypes compared to their respective untreated controls (Figure 6A). PHZ treatment induced a robust expansion of pro-inflammatory CD11c+ cell subsets in WT mice. Specifically, percentages of these CD11c+ cells expressing IL-1β (Mean Difference = 4.042; 95% CI: 1.055 to 7.028; *p* = 0.004) (Figure 6B), TNF-α (Mean Difference = 1.657; 95% CI: 0.180 to 3.133; *p* = 0.018) (Figure 6C), IL-6 (Mean Difference = 1.165; 95% CI: 0.111 to 2.218; *p* < 0.001) (Figure 6D), and IL-12 (Mean Difference = 2.357; 95% CI: 0.917 to 3.796; *p* = 0.010) (Figure 6E) were significantly decreased in Gal-3 KO mice in comparison with PHZ treated WT mice. The precise identification of cytokine-producing CD11c+ cells was guided by the gating strategy and FMO controls shown in Figure 6G. Importantly, these changes in cell frequencies were accompanied by a significant reduction in the absolute numbers of pro-inflammatory IL-1β and IL-12 producing CD11c+ cells (Table 1), further confirming that Gal-3 plays a significant role in dendritic cell activation during acute hemolytic stress.

Analysis of IL-10 expression within the CD11c+ population revealed a significant increase in WT mice following PHZ administration compared to untreated controls (Figure 6F; Mean Difference = 1.904; 95% CI: 0.015 to 3.793; *p* = 0.048). Although a similar upward trend was observed in Gal-3 KO mice, it did not reach statistical significance compared to its respective untreated group (*p* > 0.05). Importantly, no statistically significant difference was observed between PHZ-treated WT and Gal-3 KO mice regarding the frequency of IL-10 + CD11c+ cells at this time point (Figure 6F; Mean Difference = 0.358; 95% CI: −1.331 to 2.048; *p* = 1.000).

### 3.6. Absence of Gal-3 Shifts Splenic Macrophages Toward an Anti-Inflammatory Phenotype

**Figure 7:**
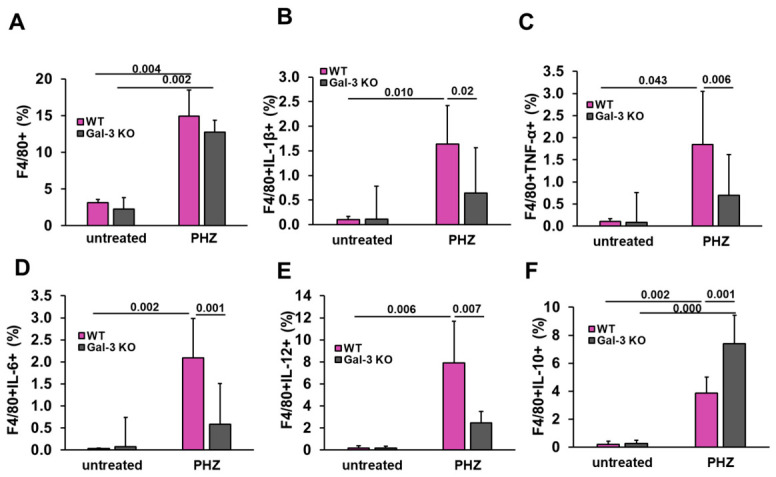
Galectin-3 deficiency shifts the splenic macrophage phenotype toward an anti-inflammatory profile during acute hemolysis. Splenic macrophage populations were analyzed by flow cytometry 48 h following PHZ administration. (**A**) Total frequency of F4/80+ macrophages in the spleen. The subsequent panels illustrate the percentage of F4/80+ cells expressing specific cytokines: (**B**) IL-1β, (**C**) TNF-α, (**D**) IL-6, (**E**) IL-12, and (**F**) IL-10. Data are presented as mean + SD. Sample sizes: untreated WT (*n* = 4), untreated Gal-3 KO (*n* = 4), PHZ-treated WT (*n* = 6), and PHZ-treated Gal-3 KO (*n* = 6). Statistical significance was assessed using one-way ANOVA with a Bonferroni post hoc test. Exact *p*-values for significant differences are indicated above the comparison brackets.

Similar to the observations in the dendritic cell compartment, PHZ administration triggered a significant expansion of the splenic macrophage population, with a marked increase in the percentage of F4/80+ cells observed in both WT and Gal-3 KO mice compared to their respective untreated controls (Figure 7A). Consistent with our observations in dendritic cells, we analyzed the functional polarization of splenic F4/80+ macrophages, which play a critical role in heme-iron recycling and systemic immune regulation during acute hemolysis. Following PHZ administration, WT mice exhibited a substantial increase in the percentage of F4/80+ macrophages expressing IL-1β, TNF-α, IL-6, and IL-12. In striking contrast, the expression of these pro-inflammatory cytokines, IL-1β (Mean Difference = 0.997; 95% CI: 0.171 to 1.822; *p* = 0.010) (Figure 7B), TNF-α (Mean Difference = 1.202; 95% CI: 0.031 to 2.373; *p* = 0.043) (Figure 7C), IL-6 (Mean Difference = 1.507; 95% CI: 0.500 to 2.513; *p* = 0.001) (Figure 7D), and IL-12 (MD = 5.423; 95% CI: 1.276 to 9.570; *p* = 0.007) (Figure 7E) was markedly attenuated in Gal-3 KO macrophages in comparison with WT mice. These results indicate that the absence of Gal-3 contributes to the transition of macrophages toward a classic inflammatory (M1-like) state in response to hemolytic stress. Post hoc analysis confirmed that for key inflammatory markers, Gal-3 KO PHZ mice clustered within a homogeneous subset with untreated control groups, further highlighting the modulatory effect of Gal-3 deficiency. Notably, while the pro-inflammatory response was blunted, we observed a significant enhancement in the anti-inflammatory capacity of the F4/80+ population. Following PHZ treatment, Gal-3 KO mice exhibited a significantly higher percentage of IL-10-producing F4/80+ cells compared to their WT counterparts (Figure 7F; MD = 3.522; 95% CI: 1.259 to 5.784; *p* = 0.001). This dual effect, the concurrent reduction in pro-inflammatory cytokines and the upregulation of IL-10, suggests that Gal-3 is an important regulator of macrophage-mediated inflammation, with its deficiency promoting a more balanced immune response.

### 3.7. Galectin-3 Deficiency Shifts the Splenic T Cell Balance Toward an Immunoregulatory Phenotype

**Figure 8:**
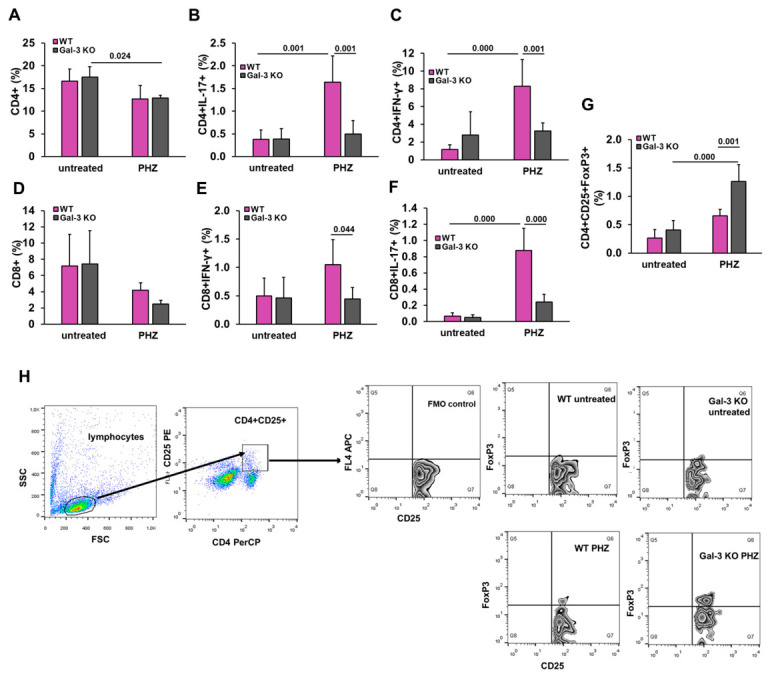
Gal-3 deficiency promotes an immunoregulatory T cell response and limits Th1/Th17 expansion during acute hemolysis. Splenic T cell populations were analyzed by flow cytometry 48 h post-PHZ administration. The graphs show the percentage of CD4+ (**A**); CD4 + IL-17+; (**B**) CD4 + IFN-γ+ (**C**); CD8; (**D**) CD8 + IFN-γ + (**E**); CD8 + IL-17+ (**F**); and CD4 + CD25 + FoxP3+ regulatory T cells (**G**). (**H**) Gating strategy and representative flow cytometry plots for Tregs. The sequence illustrates the selection of lymphocytes, followed by CD4 + CD25+ gating and final identification of Foxp3+ cells across all experimental groups. A Fluorescence Minus One (FMO) control was used to define the gating cutoffs for Foxp3-expressing CD4 + CD25+ cells. Data are presented as mean + SD (*n* = 4 animals per group for untreated mice and *n* = 6 mice per group for PHZ-treated groups). Statistical significance was determined using one-way ANOVA with a Bonferroni post hoc test, with exact *p*-values indicated above the comparison bars.

To determine the impact of Gal-3 on the adaptive immune response during acute hemolysis, we characterized the polarization of splenic T cell subsets using flow cytometry. Analysis of the total T cell pool revealed that PHZ administration led to a significant reduction in the frequency of splenic CD4+ T cells only in WT mice compared to their untreated counterparts (Figure 8A), whereas no significant changes were observed in the Gal-3 KO group following PHZ treatment. Similarly, the percentage of CD8+ T cells remained stable across both genotypes, with no statistically significant differences observed between untreated and PHZ-treated groups (Figure 8D). PHZ administration in WT mice triggered a robust inflammatory T cell response, characterized by a significant increase in the percentages of both Th1-like and Th17-like effector T cell compartments (Figure 8). Percentages of CD4+ and CD8+ T cells producing IL-17 ((MD = 1.143; 95% CI: 0.467 to 1.820; *p* = 0.001) for CD4+ and MD = 0.637; 95% CI: 0.352 to 0.922; *p* = 0.000 for CD8+) and IFN-γ (MD = 5.047; 95% CI: 1.412 to 8.681; *p* = 0.001 for CD4+; MD = 0.605; 95% CI: 0.012 to 1.198; *p* = 0.044 for CD8+) cells were significantly attenuated in Gal-3 KO mice compared to WT PHZ-treated mice (Figure 8B,C,E,F). Notably, post hoc analysis categorized Gal-3 KO PHZ mice within the same homogeneous subset as untreated controls, highlighting that the absence of Gal-3 helps maintain the immune balance during hemolytic stress. Consistent with the reduced organ damage and lower systemic inflammation observed in the knockout group, we found a significant difference in the regulatory T cell compartment. To ensure precise identification of these cells, we employed the gating strategy illustrated in Figure 8H, using FMO controls to establish accurate gating cutoffs. Following PHZ treatment, Gal-3 KO mice exhibited a significantly higher percentage of CD4 + CD25 + FoxP3+ regulatory T cells compared to their WT counterparts (Figure 8G; MD = 0.608; 95% CI: 0.260 to 0.956; *p* = 0.000). Furthermore, these shifts in cell frequencies were consistent with the absolute cell counts presented in Table 1, where Gal-3 KO mice displayed an expansion of the Treg population alongside a significant reduction in Th17 and Tc17 effector cells. This shift toward an immunoregulatory environment likely contributes to limiting collateral tissue damage during acute hemolytic crises.

These data indicate that Gal-3 modulates the splenic immune response during hemolysis by promoting a pro-inflammatory Th1/Th17 polarization while limiting the expansion of regulatory T cells (Tregs). This immunological profile in the spleen is consistent with the severity of systemic inflammation and the observed peripheral organ damage.

## 4. Discussion

The results of the present study provide a mechanistic framework for the role of Gal-3 as a significant modulator of the multi-organ stress triggered by acute intravascular hemolysis. Our findings reveal a dissociation between the primary hemolytic event and the secondary inflammatory surge. Specifically, while the degree of erythrocyte destruction, evidenced by equivalent RBC counts and hematocrit levels, was consistent across genotypes, Gal-3 deficiency was associated with attenuated hepatic, renal, and pancreatic injury. This indicates that the clinical severity of hemolytic crises may not merely reflect the initial loss of red blood cells, but is likely influenced by a Gal-3 mediated inflammatory loop [1,2,8,11]. In this context, Gal-3 appears to act as a relevant link between heme-induced cellular stress and the systemic inflammatory response syndrome, consistent with previous observations regarding the effects of Gal-3 on renal and hepatocellular injury mechanisms [21].

### 4.1. Gal-3 as a DAMP and Amplifier of Heme-Induced Systemic Inflammation and Cytotoxicity

Intravascular hemolysis leads to the abrupt release of cell-free hemoglobin, which is rapidly oxidized into labile heme, a potent DAMP that triggers sterile inflammation and direct tissue toxicity [2,9,12,29,30,31]. In this environment, our results suggest that Gal-3 may act as a relevant secondary alarmin [16,19]. Specifically, Gal-3 could function as a molecular bridge that facilitates the activation of innate immune receptors. Extracellular Gal-3 has been shown to interact with the TLR4 signaling axis, a pathway implicated in the pathogenesis of hemolysis-mediated organ injury [32,33]. Gal-3′s chimeric structure allows it to form pentamers that can cross-link cell surface glycoprotein receptors, potentially lowering the threshold for innate immune activation. This may contribute to the enhanced production of pro-inflammatory cytokines, TNF-α, IL-1β, and IL-6, which characterizes the inflammatory response observed in WT mice, but is attenuated in Gal-3 KO counterparts (Figure 6 and Figure 7). By modulating this Gal-3/TLR4 activation axis, Gal-3 deficiency may limit the downstream oxidative stress and secondary mitochondrial dysfunction associated with acute tubular necrosis and hepatocellular damage [33,34]. Thus, our data suggest that Gal-3 is not merely a marker of hemolysis, but a significant contributor to the transition from heme-induced cellular stress to systemic organ dysfunction.

### 4.2. Gal-3 as a Mediator of the Heme-Organ Injury Axis

The protection observed in Gal-3 KO mice across the liver, kidneys, and pancreas highlights the protein’s role in modulating the systemic response to hemolytic stress [20,21,35]. Our findings suggest that Gal-3 acts as a relevant link in the “heme–organ injury axis”.

In the liver, heme-induced impairment of microcirculation is a primary driver of injury. Free heme promotes the expression of adhesion molecules such as E-selectin and ICAM-1, leading to the clogging of sinusoids by activated leukocytes [14,29,35]. The reduction in ALT levels and the preservation of lobular architecture in Gal-3 KO mice (Figure 2) are consistent with Gal-3′s role in facilitating leukocyte-endothelial adhesion and hepatic stellate cell activation, as previously observed in various models of liver injury [13,36]. In the absence of Gal-3, the progression from acute oxidative stress to extensive hepatocellular necrosis appears to be attenuated, potentially limiting the severity of heme-induced liver injury [23,37].

Similarly, in the kidneys, the filtration of free heme triggers an inflammatory response, often through the activation of the NLRP3 inflammasome [9,38,39]. Given that Gal-3 has been described as a facilitator for the assembly of the NLRP3 complex [4,40,41], its absence may suppress the secretion of IL-1β and reduce the secondary wave of tubular death typically seen in heme nephropathy [25,26,39,42,43]. Our results in Figure 4 demonstrate that Gal-3 deficiency contributes to maintaining renal integrity (serum creatinine) despite the presence of hemolytic pigments.

While clinical hemolysis is a recognized cause of acute pancreatitis [17,44], the heme-pancreas axis remains less characterized than the hepatic or renal pathways. Building on our previous work [4], we show that Gal-3 is a potential bridge in this axis. While the liver and kidneys are well-established targets of heme-mediated toxicity [9,10], our findings indicate that the pancreas is also susceptible to secondary inflammatory damage in this model. The reduction in edema and preservation of acinar cell architecture in Gal-3 KO mice (Figure 3) suggest that Gal-3 is involved in the progression of pancreatic injury. Given that acinar cells are highly susceptible to TLR4-dependent activation [45], the absence of Gal-3 likely disrupts the amplification loop between released heme and the local immune response. These results expand our understanding of the pathophysiology of multi-organ stress, suggesting that Gal-3 could be considered a potential therapeutic target for modulating pancreatic complications during severe hemolytic conditions.

### 4.3. The Role of the Spleen in Heme Clearance and Regeneration

A notable finding in our study was the significantly more pronounced splenomegaly in Gal-3 KO mice compared to WT controls following PHZ-induced hemolysis, despite equivalent levels of red blood cell destruction. While splenic enlargement is often associated with disease severity, in the context of acute hemolysis, our data suggest it represents an enhanced compensatory response. The absence of Gal-3 is associated with a shift in the splenic microenvironment that may facilitate more effective extramedullary erythropoiesis [46]. Furthermore, our flow cytometry results showing an increase in regulatory T cells within the Gal-3 KO spleens suggest that this enlargement reflects a recruitment of immune subsets associated with inflammation resolution and homeostatic recovery [36]. This splenic response in Gal-3 KO mice may serve as a buffer mechanism, potentially limiting the exposure of the liver and kidneys to excessive heme and subsequent oxidative stress [47]. Thus, the loss of Gal-3 appears to shift the splenic response from a pro-inflammatory state toward a profile more focused on heme clearance and tissue repair.

Beyond direct tissue protection, our study demonstrates that Gal-3 deficiency influences the immune landscape, modulating the systemic response toward resolution. At the cellular level, this superior protection is associated with changes in the T cell and macrophage axis within the splenic microenvironment. Our flow cytometry analysis revealed a marked expansion of CD4 + CD25 + Foxp3+ regulatory T cells and a concomitant reduction in Th17 frequency in Gal-3 KO mice (Figure 8). Gal-3 is known to cross-link T cell receptors, thereby modulating the threshold for activation and survival [48,49]. In its absence, the reduced IL-6 and IL-1β milieu of cytokines observed in Gal-3 KO mice likely creates a permissive environment for Treg stability and expansion. Furthermore, the absence of Gal-3, an endogenous ligand for TLR4, may influence macrophage polarization toward a reparative phenotype. In WT mice, Gal-3 and labile heme potentially act synergistically to activate macrophages toward a pro-inflammatory phenotype, exacerbating tissue injury. Conversely, Gal-3 deficiency is associated with a more tolerogenic profile, where macrophages may be more efficient in heme scavenging and tissue repair, effectively shielding organs from oxidative stress [50]. Collectively, these cellular changes provide a plausible explanation for the resilience of Gal-3 KO mice to heme-induced damage.

Given that several Gal-3 inhibitors are currently in clinical development [51], our findings suggest that these pharmacological agents could be explored for their potential to limit organ stress during hemolytic crises, such as those seen in sickle cell disease [52]. By targeting the Gal-3/TLR4 axis, it may be possible to dampen the systemic inflammatory response and protect vital organs. Our findings indicate that Gal-3 could be a potential target for suppressing systemic inflammation and supporting erythropoietic recovery, offering a therapeutic strategy worth further investigation for managing acute hemolytic complications.

### 4.4. Limitations of the Study

Despite the clear evidence provided, several limitations should be acknowledged. First, this study utilized a chemically induced PHZ model of acute intravascular hemolysis. While this model effectively mimics acute hemolytic crises, it may not fully capture the chronic fibro-inflammatory remodeling and complex comorbidities typically seen in patients with hereditary hemoglobinopathies, such as sickle cell disease or thalassemia. Furthermore, our analysis was restricted to a single 48 h time point, which represents the acute inflammatory peak in this model; therefore, the long-term effects of Gal-3 on the resolution phase and tissue repair remain to be determined.

Second, our experiments employed a global Gal-3 knockout model; consequently, we cannot definitively distinguish the relative contribution of Gal-3 originating from circulating immune cells versus that expressed by the parenchymal cells of the liver, kidneys, or pancreas. Future studies utilizing cell-specific knockouts are required to delineate these distinct roles and clarify whether parenchymal Gal-3 contributes to organ-specific cytotoxicity through paracrine signaling. Additionally, while the sample size for certain immunological analyses was relatively modest (*n* = 4–6), the consistency of the observed phenotypic shifts across independent experimental cohorts supports the validity of our findings. Nonetheless, larger studies may be needed to further refine the detection of subtle cellular variations.

Finally, although the murine PHZ model is a well-established tool for studying sterile inflammation, inherent inter-species differences in the innate immune system suggest that further translational research in human patient cohorts is warranted to validate these findings.

## 5. Conclusions

In conclusion, our study demonstrates that Gal-3 deficiency significantly attenuates multi-organ injury during acute intravascular hemolysis, despite equivalent levels of erythrocyte destruction. Our findings identify a dissociation between the primary hemolytic event and the secondary inflammatory surge, where Gal-3-dependent signaling contributes to the development of hepatic, renal, and pancreatic stress. At the cellular level, the absence of Gal-3 was associated with an immunoregulatory shift in the splenic microenvironment, characterized by increased regulatory T cell and IL-10+ macrophage frequencies. These results suggest that Gal-3 is a significant modulator of the systemic inflammatory response in this model, positioning it as a potential target for therapeutic investigation in hemolytic conditions.

## Figures and Tables

**Table 1:** Absolute counts of splenic leukocyte populations (cells × 10^6^).

Cell Population	WT Untreated	GAL-3 KO Untreated	WT PHZ	GAL-3 KO PHZ
TOTAL SPLENOCYTES				
	7.375 ± 3.449	8.875 ± 5.483	19.750 ± 4.402	29.333 ± 10.250 ^a^
MYELOID CELLS				
CD11c+	0.530 ± 0.420	0.520 ± 0.420	4.710 ± 1.610	7.280 ± 3.160
F4/80+	0.310 ± 0.040	0.270 ± 0.160	2.990 ± 0.870	4.100 ± 1.330
CYTOKINES EXPRESSING MYELOID CELLS				
CD11c + IL-1Β+	0.040 ± 0.030	0.070 ± 0.030	1.090 ± 0.570 ^aaa^	0.380 ± 0.110 ^bb^
CD11c + TNF-A+	0.040 ± 0.010	0.040 ± 0.010	0.550 ± 0.350 ^aa^	0.270 ± 0.030
CD11c + IL-6+	0.001 ± 0.002	0.003 ± 0.002	0.344 ± 0.247 ^a^	0.132 ± 0.076
CD11c + IL-12+	0.020 ± 0.010	0.020 ± 0.010	0.710 ± 0.360 ^aaa^	0.290 ± 0.110 ^b^
CD11c + IL-10+	0.020 ± 0.010	0.050 ± 0.020	0.420 ± 0.220	0.530 ± 0.400 ^a^
F4/80 + IL-1Β+	0.010 ± 0.000	0.010 ± 0.010	0.330 ± 0.190 ^aa^	0.180 ± 0.040
F4/80 + TNF-A+	0.010 ± 0.000	0.010 ± 0.000	0.380 ± 0.310 ^a^	0.190 ± 0.040
F4/80 + IL-6+	0.002 ± 0.001	0.004 ± 0.002	0.423 ± 0.253 ^aa^	0.149 ± 0.085 ^b^
F4/80 + IL-10+	0.020 ± 0.020	0.010 ± 0.010	0.750 ± 0.260	2.250 ± 1.170 ^aa^′^bbb^
F4/80 + IL-12+	0.010 ± 0.020	0.010 ± 0.010	1.610 ± 1.030 ^aa^	0.710 ± 0.580
T CELL POPULATIONS				
CD4+	1.220 ± 0.550	1.630 ± 1.160	2.510 ± 1.100	3.780 ± 1.350 ^a^
CD8+	0.440 ± 0.080	0.500 ± 0.080	0.800 ± 0.230	0.700 ± 0.230
T CELL PHENOTYPES				
CD4 + IL-17+	0.020 ± 0.010	0.030 ± 0.030	0.330 ± 0.150 ^aa^	0.140 ± 0.110 ^b^
CD4 + IFN-Γ+	0.080 ± 0.030	0.140 ± 0.030	1.650 ± 0.680 ^aaa^	0.920 ± 0.400
CD8 + IFN-Γ+	0.030 ± 0.010	0.030 ± 0.010	0.220 ± 0.130 ^a^	0.140 ± 0.090
CD8 + IL-17+	0.000 ± 0.000	0.000 ± 0.000	0.180 ± 0.090 ^aaa^	0.060 ± 0.010 ′^bb^
CD4 + CD25 + FOXP3+	0.020 ± 0.010	0.040 ± 0.030	0.130 ± 0.040	0.360 ± 0.140 ^aaa^′^bb^

Values are expressed as mean ± SD. ^a^ *p* < 0.05 vs. corresponding untreated control. ^b^ *p* < 0.05 WT PHZ vs. Gal-3 KO PHZ; ^aa^ *p* < 0.01 vs. corresponding untreated control. ^bb^ *p* < 0.01 WT PHZ vs. Gal-3 KO PHZ; ^aaa^ *p* < 0.005 vs. corresponding untreated control. ^bbb^ *p* < 0.05 WT PHZ vs. Gal-3 KO PHZ.

## Data Availability

The raw data supporting the conclusions of this article will be made available by the authors on request.

## Abbreviations

The following abbreviations are used in this manuscript:

CD163	Cluster of Differentiation 163 (Hemoglobin scavenger receptor)
DAMPs	Damage-associated molecular patterns
Gal-3	Galectin-3
Hb	Hemoglobin
HO-1	Heme oxygenase-1
KO	Knockout
MODS	Multi-organ dysfunction syndrome
NLRP3	NOD-like receptor protein 3 inflammasome
PHZ	Phenylhydrazine
